# Supplementary figures and images for: Locally developed models improve the accuracy of remotely assessed metrics as a rapid tool to classify sandy beach morphodynamics
Source: PeerJ. 2022 May 17;10:e13413. doi: 10.7717/peerj.13413 (PMC9121867; doi:10.7717/peerj.13413)

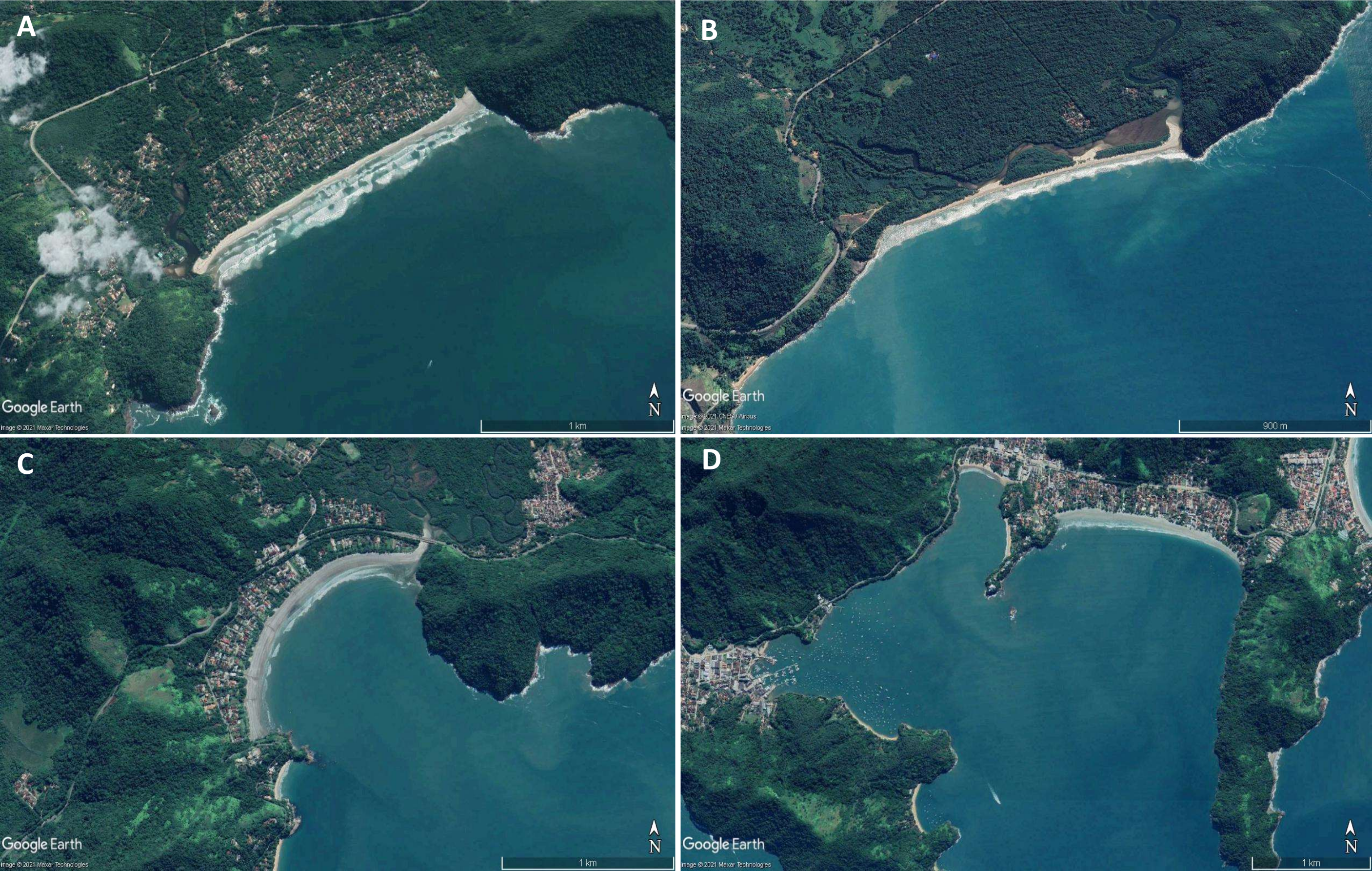

Supplement: Supplemental Information 1 — (A), (B) exposed and very exposed beaches, respectively, with little to no curvature or embayment caused by prominent headlands, and no sheltering from barrier islands. (C) moderately exposed beach, with sheltering caused by curvature of the headlands, that shelters and dissipates wave energy; and (D) sheltered and very sheltered beaches, located within embayments, with very exposure to wave action. Maps data: (A), (C) and (D) ©2021 Google, Maxar Technologies; (B) ©2021 Google, CNES/Airbus, Maxar Technologies [file peerj-10-13413-s001.png]

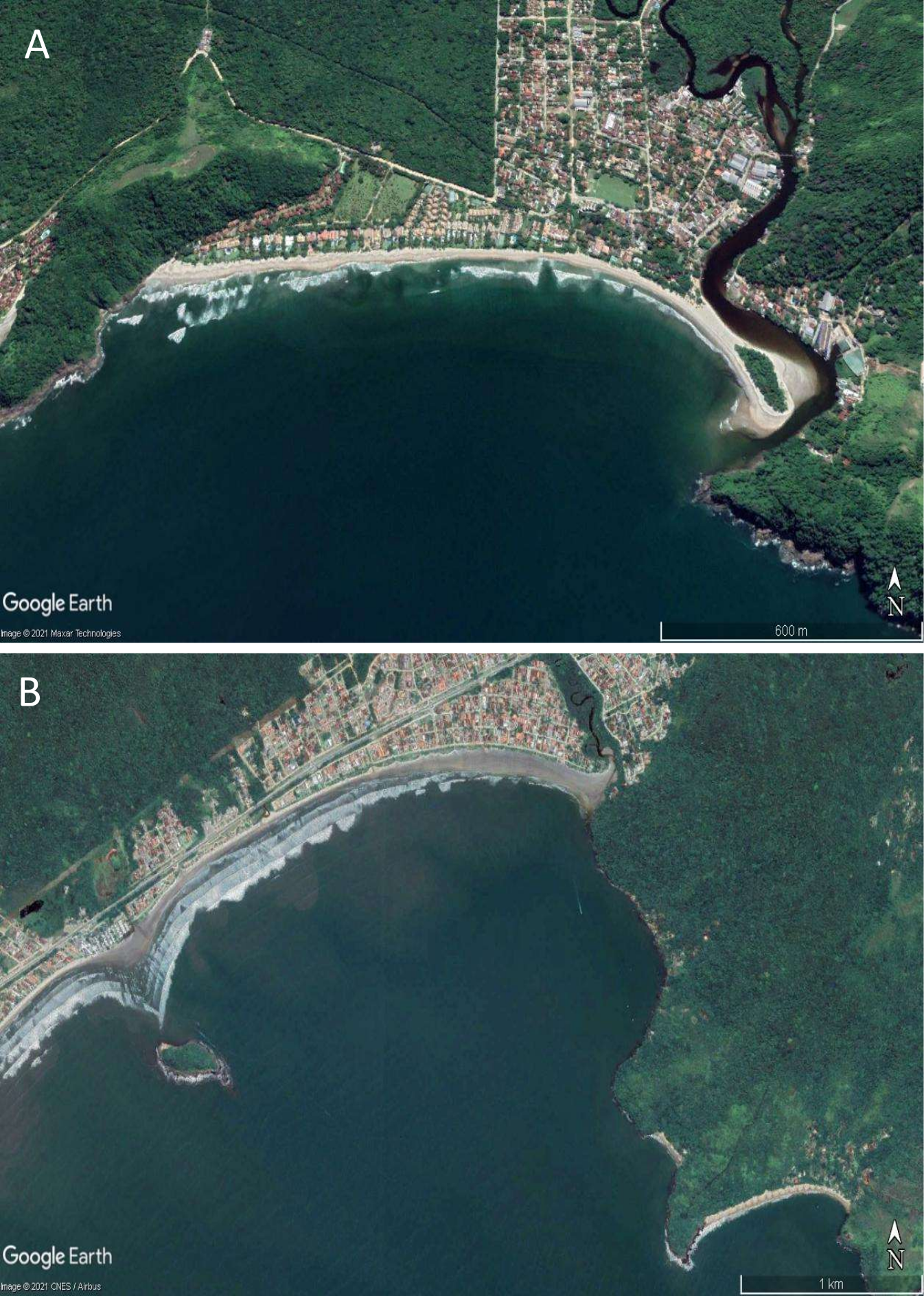

Supplement: Supplemental Information 2 — In (A) (Una beach), the right corner is a moderately exposed intermediate beach type, whereas the left corner present wave exposed reflective conditions. In (B) (Lagoinha beach), the right corner is a sheltered dissipative beach type, whereas the right corner is moderately sheltered intermediate beach. Maps data: (A) ©2021 Google, CNES/Airbus; (B) ©2021 Google, Maxar Technologies [file peerj-10-13413-s002.png]
