# Supplementary material for: Locally developed models improve the accuracy of remotely assessed metrics as a rapid tool to classify sandy beach morphodynamics
Source: PeerJ. 2022 May 17;10:e13413. doi: 10.7717/peerj.13413 (PMC9121867; doi:10.7717/peerj.13413)
Supplement: Supplemental Information 3 — Beach type is coded as: D: Dissipative, ID: Intermediate-dissipative; I: Intermediate; IR: Intermediate reflective; R: Reflective. [file peerj-10-13413-s003.docx]

**Table S1.** Summary of the in-situ characteristics of the 87 sampled sites. Beach type is coded as: D: Dissipative, ID: Intermediate-dissipative; I: Intermediate; IR: Intermediate reflective; R: Reflective.

| **Beach** | **Site** | **Intertidal Width (m)** | **Beach Index (BI)** | **Slope (s)** | **Mean grain size (ϕ)** | **Beach Type** |
| --- | --- | --- | --- | --- | --- | --- |
| Baleia | 1 | 75 | 2.553 | 0.017 | 3.07 | D |
| Baleia | 2 | 80 | 2.429 | 0.022 | 2.99 | D |
| Baleia | 3 | 60 | 2.428 | 0.022 | 2.88 | D |
| Barequeçaba | 1 | 85 | 2.551 | 0.019 | 3.37 | D |
| Barequeçaba | 2 | 80 | 2.510 | 0.021 | 3.36 | D |
| Barequeçaba | 3 | 95 | 2.523 | 0.020 | 3.39 | D |
| Barra Seca | 1 | 20 | 1.911 | 0.069 | 2.79 | I |
| Barra Seca | 2 | 15 | 1.949 | 0.068 | 3.02 | I |
| Barra Seca | 3 | 15 | 1.939 | 0.059 | 2.55 | I |
| Boiçucanga | 1 | 20 | 1.337 | 0.170 | 1.85 | R |
| Boiçucanga | 2 | 15 | 1.150 | 0.161 | 1.14 | R |
| Boiçucanga | 3 | 20 | 1.156 | 0.151 | 1.08 | R |
| Boraceia | 1 | 95 | 2.578 | 0.017 | 3.27 | D |
| Boraceia | 2 | 80 | 2.617 | 0.015 | 3.03 | D |
| Boraceia | 3 | 80 | 2.522 | 0.017 | 2.85 | D |
| Caçandoca | 1 | 25 | 1.688 | 0.066 | 1.61 | IR |
| Caçandoca | 2 | 25 | 1.604 | 0.106 | 2.13 | IR |
| Caçandoca | 3 | 25 | 1.691 | 0.069 | 1.7 | IR |
| Capricórnio | 1 | 20 | 1.275 | 0.157 | 1.48 | R |
| Capricórnio | 2 | 20 | 1.222 | 0.151 | 1.26 | R |
| Capricórnio | 3 | 20 | 1.176 | 0.193 | 1.448 | R |
| Cidade | 1 | 20 | 1.609 | 0.117 | 2.37 | I |
| Cidade | 2 | 25 | 1.708 | 0.092 | 2.36 | ID |
| Cidade | 3 | 40 | 2.147 | 0.035 | 2.47 | D |
| Domingas Dias | 1 | 30 | 1.723 | 0.075 | 1.99 | I |
| Domingas Dias | 2 | 25 | 1.786 | 0.085 | 2.59 | I |
| Domingas Dias | 3 | 25 | 1.744 | 0.088 | 2.43 | I |
| Dura | 1 | 75 | 2.516 | 0.018 | 2.89 | D |
| Dura | 2 | 90 | 2.664 | 0.014 | 3.15 | D |
| Dura | 3 | 90 | 2.549 | 0.020 | 3.46 | D |
| Fazenda | 1 | 65 | 2.365 | 0.025 | 2.87 | D |
| Fazenda | 2 | 65 | 2.431 | 0.023 | 3.13 | D |
| Fazenda | 3 | 55 | 2.361 | 0.027 | 3.07 | D |
| Félix | 1 | 25 | 1.649 | 0.055 | 1.22 | R |
| Félix | 2 | 25 | 1.667 | 0.078 | 1.82 | IR |
| Félix | 3 | 15 | 1.580 | 0.122 | 2.32 | I |
| Fortaleza | 1 | 30 | 2.055 | 0.042 | 2.405 | ID |
| Fortaleza | 2 | 30 | 2.062 | 0.048 | 2.75 | ID |
| Fortaleza | 3 | 25 | 2.024 | 0.052 | 2.77 | ID |
| Grande | 1 | 45 | 1.936 | 0.055 | 2.38 | ID |
| Grande | 2 | 40 | 1.959 | 0.051 | 2.32 | ID |
| Grande | 3 | 50 | 2.040 | 0.039 | 2.15 | ID |
| Guaecá | 1 | 30 | 2.049 | 0.044 | 2.48 | ID |
| Guaecá | 2 | 35 | 1.967 | 0.048 | 2.24 | ID |
| Guaecá | 3 | 40 | 1.863 | 0.056 | 2.05 | ID |
| Itaguá | 1 | 15 | 1.280 | 0.179 | 1.71 | IR |
| Itaguá | 2 | 20 | 1.907 | 0.052 | 2.08 | I |
| Itaguá | 3 | 25 | 1.948 | 0.065 | 2.89 | ID |
| Itamambuca | 1 | 45 | 2.255 | 0.028 | 2.52 | ID |
| Itamambuca | 2 | 45 | 1.802 | 0.074 | 2.35 | ID |
| Itamambuca | 3 | 50 | 1.735 | 0.069 | 1.87 | ID |
| Jureia | 1 | 20 | 1.353 | 0.150 | 1.69 | R |
| Jureia | 2 | 20 | 1.332 | 0.161 | 1.73 | R |
| Jureia | 3 | 25 | 1.340 | 0.140 | 1.53 | R |
| Lagoinha | 1 | 55 | 2.354 | 0.025 | 2.77 | D |
| Lagoinha | 2 | 45 | 2.200 | 0.033 | 2.59 | ID |
| Lagoinha | 3 | 15 | 1.665 | 0.106 | 2.45 | I |
| Perequê-Mirim | 1 | 30 | 2.306 | 0.028 | 2.8 | ID |
| Perequê-Mirim | 2 | 20 | 1.967 | 0.060 | 2.76 | ID |
| Perequê-Mirim | 3 | 25 | 1.882 | 0.070 | 2.65 | ID |
| Porta | 1 | 25 | 1.758 | 0.072 | 2.06 | I |
| Porta | 2 | 20 | 1.631 | 0.101 | 2.15 | IR |
| Porta | 3 | 25 | 1.630 | 0.084 | 1.79 | IR |
| Prumirim | 1 | 20 | 1.187 | 0.172 | 1.323 | R |
| Prumirim | 2 | 20 | 1.105 | 0.208 | 1.322 | R |
| Prumirim | 3 | 20 | 1.146 | 0.189 | 1.322 | R |
| Sahy | 1 | 25 | 1.670 | 0.087 | 2.04 | I |
| Sahy | 2 | 20 | 1.814 | 0.049 | 1.58 | I |
| Sahy | 3 | 15 | 1.413 | 0.103 | 1.33 | R |
| Santa Rita | 1 | 15 | 1.754 | 0.093 | 2.63 | I |
| Santa Rita | 2 | 10 | 1.776 | 0.078 | 2.33 | I |
| Santa Rita | 3 | 15 | 1.878 | 0.069 | 2.59 | I |
| Santiago | 1 | 20 | 1.709 | 0.063 | 1.61 | IR |
| Santiago | 2 | 25 | 1.464 | 0.097 | 1.41 | R |
| Santiago | 3 | 20 | 1.826 | 0.049 | 1.64 | IR |
| Tabatinga | 1 | 25 | 1.823 | 0.085 | 2.82 | I |
| Tabatinga | 2 | 25 | 1.699 | 0.084 | 2.09 | I |
| Tabatinga | 3 | 25 | 1.704 | 0.094 | 2.38 | I |
| Toque-Toque | 1 | 20 | 1.406 | 0.117 | 1.49 | R |
| Toque-Toque | 2 | 15 | 1.319 | 0.153 | 1.59 | R |
| Toque-Toque | 3 | 25 | 1.648 | 0.069 | 1.53 | IR |
| Ubatumirim | 1 | 95 | 2.674 | 0.012 | 2.83 | D |
| Ubatumirim | 2 | 40 | 2.383 | 0.024 | 2.9 | D |
| Ubatumirim | 3 | 30 | 2.178 | 0.042 | 3.19 | D |
| Una | 1 | 20 | 1.861 | 0.059 | 2.14 | I |
| Una | 2 | 30 | 1.204 | 0.135 | 1.08 | R |
| Una | 3 | 20 | 1.336 | 0.121 | 1.31 | R |
