# Supplementary material for: Locally developed models improve the accuracy of remotely assessed metrics as a rapid tool to classify sandy beach morphodynamics
Source: PeerJ. 2022 May 17;10:e13413. doi: 10.7717/peerj.13413 (PMC9121867; doi:10.7717/peerj.13413)
Supplement: Supplemental Information 4 — Beach type are coded as: D: Dissipative; ID: Intermediate-dissipative; I: Intermediate; IR: Intermediate Reflective; R: Reflective. [file peerj-10-13413-s004.docx]

**Table S2.** Morphodynamic state of the 77 beach sites used as to validate the classification model. Beach type are coded as: D: Dissipative; ID: Intermdiate-dissipative; I: Intermediate; IR: Intermediate Reflective; R: Reflective.

| **Praia** | **Site** | **Tipo** |  | **Praia** | **Site** | **Tipo** |
| --- | --- | --- | --- | --- | --- | --- |
| Vermelha do Sul | 1 | I |  | Lázaro | 2 | ID |
| Vermelha do Sul | 2 | I |  | Lázaro | 3 | ID |
| Vermelha do Sul | 3 | I |  | Camburi (Ubatuba) | 1 | I |
| Engenho | 1 | ID |  | Camburi (Ubatuba) | 2 | I |
| Engenho | 2 | ID |  | Camburi (Ubatuba) | 3 | I |
| Martim de Sá | 1 | IR |  | Puruba | 1 | IR |
| Martim de Sá | 2 | IR |  | Puruba | 2 | IR |
| Martim de Sá | 3 | I |  | Puruba | 3 | IR |
| Toninhas | 1 | IR |  | Juquehy | 1 | ID |
| Toninhas | 2 | IR |  | Juquehy | 2 | ID |
| Toninhas | 3 | ID |  | Juquehy | 3 | ID |
| Enseada | 1 | ID |  | Justa | 1 | I |
| Enseada | 2 | D |  | Justa | 2 | I |
| Enseada | 3 | D |  | Brava do Perequê | 1 | I |
| Preta do Sul | 1 | ID |  | Flamengo | 1 | I |
| Massaguaçu | 1 | R |  | Flamengo | 2 | I |
| Massaguaçu | 2 | R |  | Flamengo | 3 | I |
| Massaguaçu | 3 | R |  | Almada | 1 | ID |
| Maranduba | 1 | I |  | Almada | 2 | ID |
| Maranduba | 2 | ID |  | Brava da Almada | 1 | IR |
| Maranduba | 3 | ID |  | Brava da Almada | 2 | IR |
| Pulso | 1 | I |  | Balneário dos trabalhadores | 1 | ID |
| Pulso | 2 | I |  | Balneário dos trabalhadores | 2 | D |
| Cigarras | 1 | I |  | Toque-Toque Pequeno | 1 | IR |
| Cigarras | 2 | I |  | Toque-Toque Pequeno | 2 | IR |
| Cigarras | 3 | I |  | Toque-Toque Pequeno | 3 | IR |
| Vermelha do Norte |  | IR |  | Cocanha | 1 | I |
| Vermelha do Norte | 2 | IR |  | Cocanha | 2 | I |
| Vermelha do Norte | 3 | IR |  | Cocanha | 3 | I |
| Estaleiro do Padre | 1 | D |  | Picinguaba | 1 | I |
| Estaleiro do Padre | 2 | D |  | Picinguaba | 2 | I |
| Estaleiro do Padre | 3 | D |  | Picinguaba | 3 | I |
| Cedro | 1 | I |  | Brava do Camburi | 1 | ID |
| Vermelha do Centro | 1 | R |  | Brava do Camburi | 2 | ID |
| Vermelha do Centro | 2 | R |  | Brava do Camburi | 3 | ID |
| Vermelha do Centro | 3 | R |  | Sete Fontes | 1 | I |
| Sununga | 1 | R |  | Sete Fontes | 2 | I |
| Sununga | 2 | R |  | Sete Fontes | 3 | I |
| Lázaro | 1 | ID |  |  |  |  |
